# Why does increased microbial fermentation in the human colon shift toward butyrate?

**DOI:** 10.3934/microbiol.2024016

**Published:** 2024-05-06

**Authors:** Harry J. Flint, Petra Louis, Sylvia H. Duncan

**Affiliations:** Rowett Institute, University of Aberdeen, Foresterhill, Aberdeen, UK AB25 2ZD

**Keywords:** fermentation, human colon, butyrate, carbohydrate fiber, short chain fatty acids, theoretical modelling, colonic pH, health

## Abstract

The microbial community of the human large intestine mainly ferments dietary fiber to short chain fatty acids (SCFAs), which are efficiently absorbed by the host. The three major SCFAs (acetate, propionate, and butyrate) have different fates within the body and different effects on health. A recent analysis of 10 human volunteer studies established that the proportions of these SCFA in fecal samples significantly shifted towards butyrate as the overall concentration of SCFA increased. Butyrate plays a key role in gut health and is preferentially utilized as an energy source by the colonic epithelium. Here we discuss possible mechanisms that underlie this ‘butyrate shift’; these include the selection for butyrate-producing bacteria within the microbiota by certain types of fiber, and the possibility of additional butyrate formation from lactate and acetate by metabolite cross-feeding. However, a crucial factor appears to be the pH in the proximal colon, which decreases as the SCFA concentrations increase. A mildly acidic pH has been shown to have an important impact on microbial competition and on the stoichiometry of butyrate production. Understanding these complex interactions has been greatly aided by the refinement of theoretical models of the colonic microbiota that assume a small number (10) of microbial functional groups (MFGs).

## 1. Introduction

The short chain fatty acids (SCFA) produced by microbial fermentation of digestive residues (fiber) in the human large intestine have multiple effects on gut and systemic health [1]. This makes it important to understand what factors affect the production rates and relative concentrations of these acids in the colon. A recent paper by LaBouyer et al. (2022) [2] analyzed data on fecal SCFA concentrations from 158 volunteers at baseline (i.e., before any dietary intervention) in 10 previous human studies conducted at Aberdeen, UK. This analysis revealed some highly significant relationships between increasing total SCFA concentrations and the proportion of SCFA accounted for by branched chain fatty acids (BCFA, which declined) and by butyrate (which increased). The decline in the BCFA proportion is easily accounted for by assuming that an increased total SCFA mainly results from an increased fermentation of carbohydrate fiber. Since BCFA are only derived from fermentation of branched chain amino acids, this is consistent with the fraction of digestive residues arriving in the colon that is derived from protein decreasing with a higher fermentable fiber intake. However, it is much less obvious why the proportion of butyrate should increase. Given the importance of butyrate for colonic health [1],[3], this question needs to be considered in greater detail.

## 2. Explaining the butyrate shift

**Figure 1.**
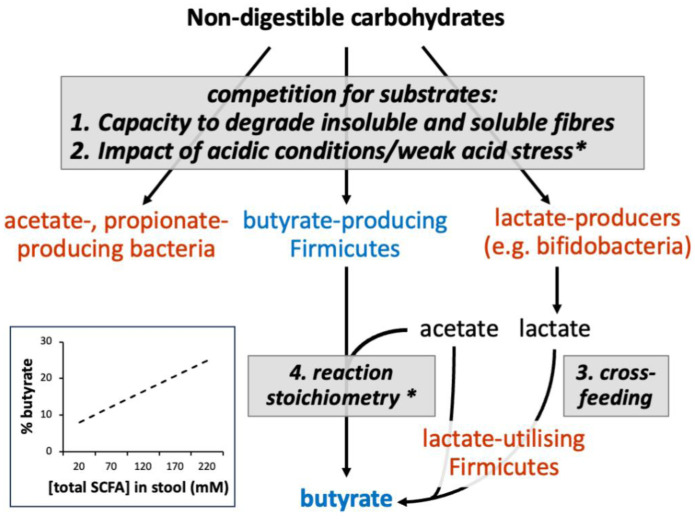
Explanations proposed for the shift towards butyrate with increasing overall fermentation in the human colon. The approximate relationship between % butyrate and total SCFA concentration reported by LaBouyer *et al*. (2022) [2] is shown in the inset (see reference for full data and statistical analyses). Numbering refers to the four mechanisms discussed in the text (* denotes those known to be impacted by pH). In our theoretical modelling [20],[25],[32], lactate producers, butyrate producers, and producers of butyrate from lactate, are defined by separate functional groups.

Why should the proportion of butyrate among acidic fermentation products of microbial fermentation increase as more digestive residue is fermented? Research from human studies, chemostat models, and isolated human colonic bacteria suggest four possible explanations for this phenomenon that are discussed below (Figure 1). In two of these, colonic pH plays the key role. It is well established that the colonic pH (especially in the proximal colon) *in vivo* decreases as the overall concentration of acids produced by fermentation increases [4],[5]. A typical pH in the proximal colon is often measured around 5.5, rising to 6.5 in the distal colon [4]–[6]. It is important to note that the highly abundant Bacteroidota (synonym Bacteroidetes) mainly produces propionate and acetate, but no butyrate, while butyrate production is a characteristic of certain genera of Bacillota (synonym Firmicutes) [7].

### 2.1. Promotion of colonic butyrate-producing or lactate-producing bacteria by fiber

Different strategies for accessing carbohydrate fibers are used by different groups of colonic bacteria. The ‘sequestration’ systems found in Bacteroidetes are equipped to capture soluble carbohydrates, whereas the extracellular enzyme systems and attachment mechanisms found in certain Firmicutes and bifidobacteria may be superior in accessing insoluble substrates such as resistant starch and plant cell wall material [8]. Butyrate-producing Firmicutes can potentially utilize a wide variety of fiber-derived carbohydrates [9]. In a carefully controlled human volunteer study, wheat bran NSP fiber was found to increase the relative abundance of certain Firmicutes, especially Lachnospiraceae, among the fecal microbiota [10]. A follow-up study conducted *in vitro* at a controlled pH of 6.5 identified butyrate-producing Firmicutes that became enriched by association with insoluble wheat bran [11].

Another human study showed that reducing dietary fiber (and total carbohydrate) intake decreased the absolute numbers and relative abundance of butyrate-producing Firmicutes related to *Roseburia* and *E. rectale* [12]. This suggests that some butyrate producers could be directly promoted by certain fiber sources. Meanwhile, there is good evidence that lactate-producing Actinobacteria, especially bifidobacteria, are promoted by carbohydrate fibers, including prebiotics [13].

### 2.2. Impact of pH on microbial competition

A slightly acidic pH is known to have differential effects on the bacterial growth rates [14]. Human colonic anaerobes tested in pure cultures showed a range of pH sensitivities in the presence of physiological concentrations of fermentation acids, with *Bacteroides* strains among those showing the greatest sensitivity to a pH of 5.5, whereas many butyrate-producing Firmicutes showed a greater tolerance [15]. This must reflect differences in physiological responses to pH changes and a sensitivity to weak acids [16],[17]. Studies examining communities of human colonic bacteria maintained in anaerobic, pH-controlled, continuous flow fermenters supplied with soluble fibers have shown that the Gram-negative *Bacteroides* dominate the community at near neutral pH values around 6.5 [18]–[20]. *Bacteroides* spp. mainly produce acetate and propionate, which were the major metabolites detected at this pH; however, lower pH values resulted in a shift towards either butyrate or lactate. A suppression of *Bacteroides* growth at a lower pH appears to help species more tolerant of acidic pH levels, including butyrate-producing Firmicutes and lactate-producing bifidobacteria, to compete for the carbohydrate substrates supplied, thus increasing their relative abundance in the community [18]–[20]. A negative correlation between the fecal pH and the relative abundance of butyrate-producing *Roseburia*-related bacteria has been reported *in vivo* [2].

### 2.3. Production of butyrate from lactate by metabolite cross-feeding

Certain Firmicutes bacteria (*Anaerostipes hadrus, A. caccae, Anaerobutyricum hallii, A. soehngenii*) have the ability to convert lactate and acetate into butyrate [21],[22]. Provided that the pH is not too low, this results in an efficient conversion within the microbial community of lactate (e.g., produced by bifidobacteria and lactobacilli) into butyrate [20],[23]. It is well established that the relative abundance of bifidobacteria can be promoted by many different dietary fibers, including prebiotics, and by an acidic pH, thus potentially increasing the supply of lactate in the colon.

### 2.4. pH dependent changes in the stoichiometry of butyrate formation

**Figure 2.**
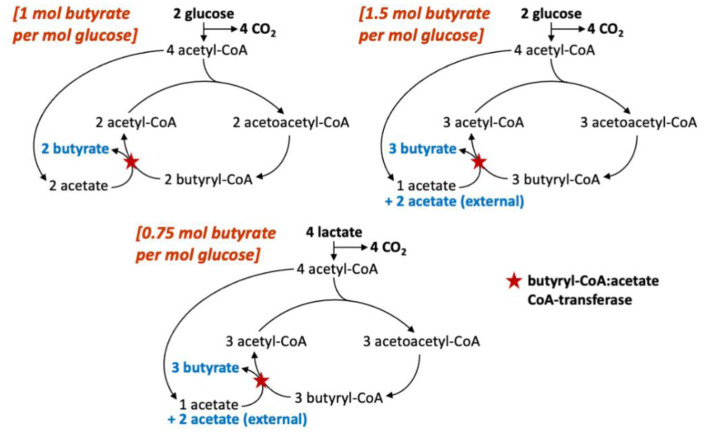
Variable stoichiometry of butyrate formation. Many of the butyrate-producing bacteria in the human colon take up external acetate via the CoA-transferase reaction, increasing the yield of butyrate from fermentation. Butyrate yields with no net acetate uptake (left) and uptake of 1 mol acetate per mol glucose (right) are shown here. Acetate uptake and hence butyrate yield increases with lower pH [7],[25]. Butyrate yield from fermentation of lactate and acetate is shown at the bottom. These highly simplified schemes show all carbon going to butyrate or carbon dioxide. In reality, some carbon is often diverted into other products such as formate and lactate. We do not show hydrogen or water here, but net acetate utilization can result in less hydrogen being formed by butyrate producers such as *Roseburia* spp. as shown previously [7].

In the predominant Firmicutes bacteria that are major producers of butyrate, the final step in butyrate formation is achieved via the enzyme butyryl-CoA:acetate CoA-transferase, which utilizes acetate and butyryl-CoA as substrates (Figure 2) [7]. This often involves the net uptake of acetate, such that the carbon in butyrate is partly derived from carbohydrate fermentation and partly from acetate [24]. In isolates of *Roseburia* spp. and *Faecalibacterium* spp., it has been shown that a slightly more acidic pH leads to a greater uptake of acetate and a greater production of butyrate for every mol of glucose fermented [7],[25]. In a study that involved batch culture incubations with mixed faecal microbiota, the composition of the microbial community was compared with the metabolites produced [26], taking the phylogenetic distribution of the relevant fermentation pathways into account [7]. At a given % of potential propionate producers within the community, there was no impact of the initial pH (5.5 or 6.5) on the % propionate among the SCFA products. In marked contrast, for any given % of butyrate producers within the community, there was a higher butyrate % among the SCFA products at a pH of 5.5 compared to at a pH of 6.5 [26]. This strongly suggests that more acidic conditions are likely to boost butyrate production in the mixed community *in vivo* simply because of a shift in the stoichiometry of butyrate formation.

## 3. Other factors that can influence growth and metabolism of butyrate-producing bacteria in the colon

The aforementioned explanations should not be regarded as strict alternatives, and all four mechanisms may play a role. It is not easy to distinguish the impact of a decreasing pH (a consequence of fermentation) on microbial competition (2, above) from the possible selective promotion of butyrate producers by certain fiber sources (1, above). Indeed, the decrease in the colonic pH may be largely responsible for the promotion of butyrate by fiber. It is also likely that these different mechanisms will vary in importance between individuals, which is influenced by diet choices and differences in the microbiota composition (in particular, the representation of butyrate producers, bifidobacteria, and lactate utilizers (Figure 1)).

Individuals also vary in the representation of methanogenic archaea within their gut microbiota. Methanogens, especially *Methanobrevibacter smithii*, are hydrogenotrophs whose activity can depress the concentration of hydrogen ([H_2_]) resulting from fermentation. Recent work indicated that [H_2_] in the colon contents had the potential to influence the metabolism and populations of butyrate-producing bacteria within the microbial community [27]. Responses differed between major butyrate-producing species *Faecalibacterium prausnitzii*, *Roseburia intestinalis*, and *Eubacterium rectale*, depending in part on whether they possess a ferredoxin hydrogenase that enables the production of hydrogen as an electron sink. This suggests that colonic [H_2_] may have an important influence on growth and the metabolism of some butyrate producers by shifting the metabolism toward a higher relative production of fermentation acids at the expense of hydrogen. It is also suggested that high fiber intakes will tend to increase H_2_ production by fermentation, with the hydrogenotrophs within the community (acetogens, sulfate-reducing bacteria and methanogenic archaea) failing to utilize all of the H_2_ produced. Methanogenic individuals have been reported to show lower fecal butyrate concentrations as compared to non-methanogenic individuals [27],[28], although differences in gut transit could also be a factor here. It can also be noted that the supply of vitamins and trace nutrients have the potential to alter populations of individual butyrate producers, many of which lack the ability to synthesize essential vitamins [29]. Moreover, butyrate-producing species widely vary in their oxygen sensitivity and responses to low oxygen concentrations [30]. Oxygen consumption accompanying butyrate oxidation by epithelial cells may play a particularly important role in determining the microenvironment of the intestinal mucosa [31].

## 4. Theoretical modelling of microbial metabolism in the colon

With such a complex ecosystem, it is important to critically examine the explanatory hypotheses. For example, are the differences in the growth rate and pH sensitivities observed between isolated strains sufficient to explain the pH-driven shifts in the microbiota composition and SCFA ratios that occur within the complex microbial community? To address this, some years ago, a theoretical model was developed to represent the human colonic microbiota and its major metabolic products. This model postulated 10 microbial functional groups (MFG) that differed, among other things, in the substrate preferences, metabolic end products, and pH sensitivities, and made use of experimental data on growth rates and pH sensitivities obtained from isolated bacteria [25]. The model has remarkably proved successful in predicting the transition seen in the microbial community and metabolite profiles between mildly acidic and near neutral pHs in our chemostat studies [18],[20].

The same theoretical model has recently been greatly refined [32] to make it relevant to the interpretation of data obtained from *in vivo* studies. Refinement required the introduction of multiple gut compartments, gut transit, mucosal absorption of SCFA and water, and a link between the local SFCA concentration and the gut luminal pH. We can note that, retaining our assumptions on the differential sensitivities of the MFG to the pH, the refined model predicted an increase in the butyrate proportion, with an increasing total concentration of fermentation acids within the feces [32]. Importantly, this modelling helped to establish that the explanations for the observed ‘butyrate shift’ given above are consistent at a rigorous quantitative level, with the experimental data from multiple human studies reported by LaBouyer *et al*. [2].

## 5. What are the consequences of the ‘butyrate shift’ for the host?

Is the ‘butyrate shift’ that accompanies increased fermentation simply an unavoidable consequence of the fundamental biochemistry and microbial ecology of the large intestinal microbiota? Or could this phenomenon be modulated by different host physiological responses? If the latter is true, then this implies that there is a net benefit to the host from the ‘butyrate shift’. The pH within the proximal colon is broadly under the control of the host through the secretion of bicarbonate that tends to neutralize fermentation acids and provides buffering [33]. Therefore, this ability to regulate the colonic pH should allow the host some control over the supply of butyrate relative to that of the other fermentation acids. Since the colonic epithelium uses butyrate as its preferred energy source, an enhanced butyrate supply in healthy individuals can contribute to colonic health by maximizing the trophic effect of microbially-produced butyrate in maintaining a healthy mucosal barrier function [1],[3],[34]. This might explain why the pH is often allowed to drop into the mid-pH 5 range in the proximal colon [5]. AN adequate butyrate supply is considered an important factor in the prevention of colorectal cancer through its multiple influences on the physiology of epithelial cells [35]. Furthermore, Roediger [36] proposed that inflammatory bowel disease could be considered an energy deficiency condition, as the SCFA (especially butyrate) supply is often severely disrupted. We now know that this disruption is accompanied by major changes in the intestinal microbiota, especially a loss of butyrate producing Firmicutes [37]. While the factors involved are complex, and an interplay with the host's immune system is crucial, departure from the normal balance of fermentation and the energy supply remains an important feature of the pathogenesis of these conditions [31]. Indeed, the fecal pH can become too acidic in severe colitis, to the point of acidosis, where production of both propionate and butyrate can become compromised and lactate accumulates, this emphasizing the very fine balance that is required for healthy gut function [20],[38]. We should also note that high rates of fermentation can have a negative health impact for some individuals, especially those suffering from inflammatory bowel diseases and irritable bowel disorders [39]. In conclusion, a better understanding of the impact of pH-dependent shifts in fermentation patterns within the human colon is very important if we are to properly understand the role of diet and the gut microbiota in overall health. There is a need for more comprehensive human volunteer studies that combine fecal microbiota profiling with dietary intake data and reliable measurements of fecal (or colonic) pH, SCFA, and breath hydrogen. These would include new dietary intervention studies that involve fiber sources. For a more complete picture, it would also be important to try to monitor gut transit in such studies. Careful data analyses, together with theoretical modelling, should then reveal the importance of inter-individual microbiota variation and factors such as pH, dietary fiber, and transit in determining SCFA and butyrate outputs. The butyrate producing capacity can be estimated from DNA sequence data and related to the examined butyrate concentration [26]. In addition, studies on isolated butyrate-producing bacteria, including work with a defined consortia, could help us to understand the ecology of these organisms and explain their behavior within the complex community *in vivo* [27],[29],[30].

## Disclosure of potential conflicts of interest

No potential conflicts of interest were disclosed.
